# Supplementary material for: Are carbon emissions trading and green financial instruments synergistic? -Comprehensive quantitative research based on content analysis
Source: PLoS One. 2024 Mar 7;19(3):e0298601. doi: 10.1371/journal.pone.0298601 (PMC10919601; doi:10.1371/journal.pone.0298601)
Supplement: S1 Data — (ZIP) [file pone.0298601.s001.zip › Data/地级市Z_F工作报告.zip!/║┌┴·╜¡/╞▀╠¿║╙╩╨/└┤╫╘╣1⁄2╓┌║┼í╛┬φ┐╦╩2╛▌═°í┐_2010.docx]

各位代表：

　　现在，我代表七台河市人民政府，向大会作政府工作报告，请予审议，并请列席会议的同志提出意见。

　　一、2009年工作回顾

　　2009年，是新世纪以来我市经济社会发展极不平凡的一年，也是我们砥砺奋进、经受严峻考验的一年。在省委、省政府和市委的正确领导下，在市人大、市政协的监督和支持下，我们深入贯彻落实科学发展观，认真贯彻落实胡锦涛总书记视察黑龙江时的重要讲话精神，万众一心，攻坚克难，圆满完成了市九届人大五次会议确定的各项任务，取得了困境中跳跃式发展的新成就。

　　——全力以赴保增长，经济跨上新台阶。在国际金融危机严重动荡、国内外形势严峻复杂的情况下，我们认真落实国家和省“保增长、扩内需、调结构”的决策部署，出台了扶持焦化企业、木制品企业和中小企业发展等一系列政策措施，努力化解各种矛盾，加大工作落实力度，实现了主导产业不下滑、骨干企业不倒闭、经济跨上新台阶。预计全市生产总值234亿元，站到200亿元台阶之上，增幅全省第一。全口径财政收入38.5亿元，人均财力跃居全省第二位。加强“三农”工作，粮食产量达到12.9亿斤，再创历史新高。加强生产要素调节，支持具备安全生产条件的煤矿和焦化企业满负荷生产，规模以上工业增加值实现116.3亿元，突破百亿元大关，增幅全省第一。加大投资力度，扩大消费需求，拓展开放领域，城镇固定资产投资135亿元，增长50%；社会消费品零售总额43亿元，增长19.4%；外贸进出口总额4380万美元，增长20%。经济保持了平稳较快发展。

　　——狠抓项目调结构，发展迎来新希望。我们立足当前，把扩大投资作为拉动增长的重要措施，严格大项目包保责任制，全力推进新一轮投资扩张，新建和改扩建项目154个，形成一批新的产能和税源主体。我们着眼长远，努力培育跳跃式发展新优势。加快基础设施建设，总投资57.3亿元的依七高速、鹤大高速七台河段、七勃公路改扩建、庆云变电站、九龙水库、汪清水库消险增容六个重大项目开工建设，是我市重大基础设施项目投资最大、数量最多、进度最快的一年，终结了我市没有自建高速公路的历史。七密铁路、新铁路客运站等项目进入实质性建设前期阶段。经济开发区具备了承接大项目的能力。推进发展方式转变，不断壮大主导产业，制定了煤化工产业发展规划，形成了焦化产业集群优势，全力推进循环经济。着力优化产业结构，建立经济转型推进机制，超前谋划和扶持非煤替代主导产业，出台了木制品产业发展规划和优惠政策，强力推进“一号工程”，木制品、新型建材、农畜产品加工、机械制造等优势产业快速发展。产业项目、重大基础设施、优势产业，必将成为我市发展后劲之所在、力量之所在。

　　——优化环境抓招商，开放迈出新步伐。我们积极转变政府职能，营造优良的发展环境，出台了招商引资奖励办法，完善了项目库，认真实行招商引资目标责任制，引进千万元以上项目82个。积极扩大对外开放，制定了《促进全市外经贸发展的意见》，对外贸易创历史最好水平。成功举办、承办了家具节、矿山物资交易博览会和国际乒乓球男子争霸赛。对外交往日益扩大，积极组织参加各种展会，加强国际经贸科技文化交流，开通直达俄罗斯国际客货运输线路，首次以外国城市命名了道路。先后赴山西等五省六市考察学习，与阳泉结为友好城市，与东北东部十二市（州）结为区域合作城市，城市品牌形象和影响力明显提升。

　　——审时度势抓机遇，改革实现新突破。我们认真研究政策，深刻剖析市情，抢抓机遇，用足用好政策。以“城乡一体化建设推进年”为载体，着力破除城乡二元结构，在全省率先统一了城乡户籍、低保补差等8项政策标准。投资3亿多元，为乡镇和村新建了标准化卫生院（所）、文化站（室）、寄宿制学校及农民新居，新修了43条通村公路，解决了46个村屯饮水安全问题。在全省率先规范机关事业单位津贴补贴、实施教师绩效工资。成功调整了行政区划，市区面积扩大到3646平方公里，拓展了城市发展空间。搭建“政银企会商网络平台”，各项贷款余额比年初增加35.1亿元，是金融机构支持地方力度最大的一年。成功与韩国产业银行签订合作协议。坚持不懈地做对上争取工作，获国家经济转型和省低碳经济试点市政策支持项目96个、15.7亿元，争取到省煤炭资源详查项目1.79亿元资金支持。改革攻坚的实质性突破，为经济社会协调发展提供了制度保障。

　　——建管结合提层次，城乡呈现新面貌。我们以创建国家园林城市为目标，扎实开展“山水园林城市建设年”、“城乡环境治理年”活动。强化规划控制引导，编制绿化、照明、滨水城市等一系列规划。投资6亿多元，推进城乡绿化、基础设施、滨水景观、“三供四治”、城乡环境治理五大工程。大力开展学阳泉、学长治、学朔州活动，开展了规模空前的造林绿化工作，投资规模、参与人数、造林面积均实现了历史性突破，新增绿地面积273.4公顷，建成区绿化覆盖率、绿地率、人均公园绿地面积均超过国家园林城市绿化标准。开工建设了人民公园、体育会展中心，完成了山湖路等主干道路改造工程和仙洞山环山路等路灯安装工程。新建万宝荷花园、仙洞山艺苑、倭肯河清园等景观工程，提升了城市品位。“三供四治”成效显著。全面加强节能减排，落实重点节能减排项目11个，完成省控节能减排指标。加强水源地保护，强化环境执法，城乡面貌和生态环境明显改善。

　　——统筹兼顾惠民生，生活再上新水平。我们大力发展各项社会事业，优先发展教育事业，加大教育投入力度，新建、改扩建5所学校，完成校舍安全工程普查和鉴定工作，强化教师队伍建设，七台河技师学院挂牌成立。三级科技企业孵化器建设进入新阶段，先进实用技术在循环经济中得到广泛应用。城乡文化设施进一步完善，社区建设全面加强，群众性文化活动丰富多彩，精神文明创建活动深入开展。公共卫生服务体系进一步健全，改造扩建8所医院，新建2个标准化社区卫生服务中心，政府为群众购买18项公共卫生服务，市医院晋升为三甲医院。有效控制了手足口病、甲型H1N1流感等重大传染性疾病。千方百计扩大就业，实施零就业家庭专项援助和促进返乡农民工就业专项行动，城镇登记失业率低于省控指标1.2个百分点。着力提高社会保障能力，提高重点优抚对象、遗属和“三无”人员、农村医疗救助、城市低保户供热、社区工作人员补贴标准。将11083名“五七工”、“家属工”纳入养老保险统筹范围。新农合参合率达96%。全年用于支持社保和就业资金8.3亿元，占财政支出总额的19.3%，是“十一五”以来民生投入最大、惠及人数最多的一年。建成市儿童福利院和流浪儿童救助保护中心。改造农村泥草房4610户，新建廉租房633套，棚改项目完成主体工程。城镇居民人均可支配收入、农民人均纯收入均实现两位数增长，分别达到14120元、5562元。城乡居民储蓄存款余额162.4亿元，增长17.7%。

　　——突出重点保安全，稳定取得新成效。我们坚持安全第一、预防为主、综合治理方针，深入开展以煤矿为重点的安全生产“三项行动”，各类事故起数、死亡人数分别下降10.2%和46.4%。连续28年无森林火灾。强化社会治安综合治理，群众安全感普遍增强。出色完成“大冬会”、“国庆60周年”等重大节庆安保任务。畅通群众诉求渠道，设立信访维稳资金，开展化解信访积案“百日会战”和领导干部大接访活动，信访形势总体平稳，社会和谐稳定。

　　——依法行政强基础，作风上有新转变。我们认真执行市人大及其常委会的决议决定，自觉接受市人大及其常委会的法律监督，主动接受市政协和各民主党派的民主监督，注重加强行政监督、审计监督和社会舆论监督，办理人大建议和政协提案189件。深入开展学习实践科学发展观活动，扎实推进学习型机关建设。加大创建优良经济发展环境力度，扩大政务公开范围，强化效能监察和行政问责，办事效率得到提高。

　　过去一年，人事、统计、审计、外事、侨务、食品药品、老龄、气象、地震、人防、地方志、档案、民兵、预备役等工作得到加强，军政军民团结进一步巩固，司法、民族宗教、新闻出版、广播电视、人口和计划生育、红十字、妇女儿童、残疾人等工作也取得长足进步。中省直单位全力支持地方建设，为全市经济社会发展做出重要贡献。

　　各位代表，经过历届市委、市政府和全市人民多年的不懈奋斗，一个奋发进取、充满活力的七台河，正在被越来越多的人们所关注、所认同。去年在困难形势下取得的成绩，是省委、省政府和市委正确领导的结果，是市人大、市政协积极支持的结果，是全市人民同心同德、顽强拼搏的结果。在此，我代表市人民政府，向全市各族人民，向所有关心、支持七台河发展的各界人士，表示诚挚的感谢！

　　我们也清醒地看到，我市经济社会发展仍然存在不少矛盾和问题，政府工作还有一些缺点和不足。一是受国际金融危机外部环境与我市经济结构不够合理、发展方式转变不快、企业创新能力较弱等深层次矛盾的双重影响，经济下行压力加大。二是资源约束相对加剧，生态环境比较脆弱，节能减排任务艰巨。三是安全生产形势依然严峻，安全生产工作压力很大。四是一些涉及群众切身利益的问题有待进一步解决，社会保障水平不高。五是政府职能转变还不到位，社会管理和公共服务比较薄弱，一些政府工作人员大局意识、责任意识、服务意识不强，形式主义、官僚主义问题比较突出，发展环境仍需改善。

　　我们一定要以高度负责的精神，重视解决存在的问题，扎扎实实地做好政府各项工作，不辜负人民的期望和重托。

　　二、2010年工作总体部署

　　今年是实施“十一五”规划的最后一年，是实现跳跃式发展三年目标的决战之年。做好今年工作，对于进一步有效应对国际金融危机，为“十二五”规划启动实施创造良好条件至关重要。

　　今年我市经济社会面临的形势依然严峻，后金融危机影响仍然存在，世界经济复苏的基础还不稳固，不确定因素较多。但发展环境总体好于去年已成共识。特别是金融危机的冲击没有动摇我市发展的基础，我市经济社会发展的基本面没有改变。国家继续实施积极的财政政策和适度宽松的货币政策，出台进一步振兴东北地区等老工业基地政策，有利于我们调整优化产业结构、加快转型步伐、完善基础设施。我市集循环经济、经济转型、低碳经济三个试点市的政策支持，一批新建成项目将发挥效益，为经济增长提供有力支撑。我们既要增强忧患意识，把困难估计得充分一些，把措施考虑得周密一些，努力把各种不利影响降到最低；更要增强机遇意识，坚定信心，凝聚力量，力争经过新一轮调整，使综合经济实力有一个大提高、发展方式有一个大转变、城市竞争力有一个大提升。

　　政府工作的基本思路和总体目标是：以邓小平理论和“三个代表”重要思想为指导，深入贯彻落实科学发展观，全面贯彻党的十七大、十七届四中全会和省委十届十次全会、市委七届八次全会精神，立足保持经济平稳较快增长，加快转变发展方式和调整经济结构，提高可持续发展能力；推进改革开放和自主创新，增强经济社会发展活力和动力；全力保障和改善民生，抓好安全生产，维护社会和谐稳定，全面完成“十一五”规划和跳跃式发展三年目标。

　　综合考虑各种因素，今年全市经济社会发展的主要目标是：地区生产总值增长15%、力争达到20%，财政一般预算收入增长11%、力争达到15%，全社会固定资产投资增长35%以上，城镇居民人均可支配收入增长11%以上，农民人均纯收入增长8%以上，万元GDP能耗下降6.6%。

　　要实现今年经济社会发展的主要目标，必须坚持强基础、优环境，壮大主导产业和骨干企业，支持中小企业加快发展，加强产业项目和城市基础设施建设，提升经济社会发展基础支撑，增强经济发展承载力。必须不懈抓转型、调结构，大力发展循环经济、低碳经济，加快发展替代产业，积极培育战略性新兴产业，增强可持续发展能力。必须突出提质量、增效益，推动产业优化升级，鼓励资源精深加工，加快自主创新和科技成果转化，提高经营管理水平，实现速度、质量、结构、效益相统一。必须始终扩内需、惠民生，增加群众收入，提高保障能力，稳定消费预期，激活民间资本，大力发展社会事业，让人民群众共享改革发展成果。必须全力保稳定、促和谐，全面加强安全生产，认真化解社会矛盾，维护社会公平正义，促进社会和谐进步。

　　三、2010年主要任务

　　今年的政府工作，要以促进经济平稳较快发展为主线，着力做好以下重点工作：

　　（一）以保持经济平稳较快发展为首要任务，着力扩大投资和消费需求。全面落实国家和省出台的一系列政策措施，积极引导扩大投资需求和消费需求，以投资带消费，以消费促增长。

　　加大投资力度和规模。突出工业、基础设施、生态环境和民生领域，重点推进47个大项目，争取13个项目建成投产。突出抓好工业项目投资。支持七煤公司搞好新建矿井建设，启动国有矿区深部煤炭详查项目。推进七煤公司铁东矸石电厂、庆云变电站和城市供电环路工程建设。完成两个98万吨焦炉续建工程，加快焦炉煤气制甲醇项目整合。力争完成宝泰隆灰渣处理等3个新型建材项目。突出抓好重大基础设施投资。完成依七高速、七勃公路改扩建、体育会展中心工程建设。开工建设公路客运中心、新铁路客运站。加快七密铁路前期工作。谋划七宝、七密一级公路改造项目。支持桃山水库二期工程加快建设。高标准推进九龙水库、汪清水库消险增容工程建设。加快推进开发区基础设施和标准化厂房建设。突出抓好民生项目投资。完成污水处理厂、新兴区垃圾处理场工程。加快推进“三供四治”、保障性住房、棚户区、农村泥草房改造等项目建设。

　　千方百计扩大消费需求。努力增加居民收入，完善保障机制，提高消费能力，稳定消费预期。拓宽消费领域，改善消费环境，搞好消费维权。促进房地产市场稳定健康发展，满足多层次住房消费需求。大力实施“万村千乡”市场工程，全面推进“家电下乡”。大力发展社区商业、物业、家政等服务性消费和文化、旅游、休闲、体育健身等消费热点。

　　（二）以结构调整为主要途径，加快推进发展方式转变。按照“三个试点市”总体要求，围绕保增长、促升级，切实增强创新能力，加快由资源驱动向要素驱动、创新驱动转变。

　　推进主导产业优化升级。积极推进煤矿安全技术改造和标准化建设，鼓励有实力的企业在境内外收购、兼并、重组煤矿，赴俄远东地区开发煤炭资源，稳定原煤产量。充分发挥焦化产业优势，用高新技术改造提升传统产业。进一步整合焦化资源，扩大规模，增加品种，延长产业链条，提高附加值，叫响“东北焦化之都”品牌。支持煤炭综合利用，积极发展新型建材和劣质煤、洗中煤、煤矸石发电项目。

　　推动战略性支柱产业加快发展。大力发展木制品、食品、机械制造等优势产业和体育会展、高新技术产业，推动支柱产业多元化。培育壮大木制品产业集群，发展集成材、五金、玻璃、油漆、包装物等配套产业，提高产业集中度。以大企业、大园区、大市场、大展会为平台，加大企业联合重组力度和引进知名企业力度。改造提升传统服务业，加快发展现代服务业，做大做强旅游业，以园区建设推动现代物流业发展。

　　积极培育发展新兴产业。抢抓国家发展战略性新兴产业机遇，把科技含量高、市场前景好的新能源、新材料、新型环保产业作为新的经济增长点，加大扶持力度，促进加快发展。突出抓好风电、生物质能源、煤层气开发利用、石墨制品、精细化工材料以及节能项目和节能产品开发。

　　加快发展低碳绿色经济。按照“低能耗、低污染、低排放”的低碳经济发展模式，坚持将资源开发、产业发展融于生态建设之中，围绕循环经济和资源型城市经济转型，大力发展节能环保产业。推广工业、建筑、交通和公用事业节能，积极引进节能、节水和综合利用技术改造项目。加强再生资源回收利用。

　　（三）以加大扶持“三农”力度为重点，加快推进城乡一体化进程。坚持公共财政向农村倾斜，基础设施向农村延伸，城市文明向农村辐射，社会保障向农村覆盖，全面加快城乡一体化进程。

　　大力发展现代农业。提高农业产业化、规模化、标准化水平，引导土地经营权集中连片流转。稳定粮食生产，合理调整种植结构，扩大特色作物种植面积。积极发展养殖大户、标准化小区和专业村。扶持发展各类专业合作组织。提高农村劳动力转移组织化程度，多渠道增加农民收入。

　　加快发展县（区）域经济。坚持把发展县（区）域经济与推进城乡一体化相结合、与新农村建设相结合、与小城镇规划建设管理相结合，加大对县（区）域经济发展的支持力度，抓好中低产田改造、小流域治理、病险水库除险加固工程建设。支持财源项目建设。强力推进县（区）域工业化、农业产业化，加快工业集中区和农畜产品基地建设，因地制宜发展二、三产业。

　　积极推进小城镇建设。完善小城镇发展规划，发挥城市对农村的辐射带动作用，搞好农民集中居住区建设和城中村改造，完善配套设施，加强农村公共服务能力建设，提高农村教育、卫生、文化服务水平。加快农民向小城镇集聚进程，统筹城乡社会管理，搞好农村社区化建设试点，培育新型农民。着力打造特色突出的工业重镇、商贸大镇、经济强镇，力争把中心河乡、双河镇建成小城镇示范镇。

　　（四）以创建国家园林城市为契机，推进生态文明建设。对照国家园林城市标准，集中力量，攻坚克难，完成申报的各项准备工作，加快建设适宜人居、利于创业，城市与山水交相辉映、人与自然和谐相融的国家园林城市。

　　高标准、高质量修订完善城市规划。坚持科学规划、适度超前、突出特色、以人为本原则，搞好新一轮城市规划和土地总体利用规划修编，完善城市规划区控详规划，完成棚户区、城中村改造规划及乡镇、村屯规划编制工作。

　　全面提升造林绿化水平。以“十大绿化工程”为载体，继续开展全民造绿活动，突出抓好“三山两湖”节点绿化和七勃路万宝河段、杨扬大街东延等绿化带建设，规划建设桃山湖、万宝河等沿湖、沿河滨水景观带。加大城区道路改造。完成人民公园建设，新建种畜场金河森林公园和茄子河区、新兴区公园。积极推进水库坝下治理开发。扎实推进村屯、公路、小区、庭院、库渠堤坝绿化和荒山荒地造林。

　　全力抓好“三供四治”工程。完成风采路、桃山六井、茄子河镇供水管线建设，实施棚改新区、经济开发区等供热管网工程，加快七煤集团煤气公司改造扩能，做好汪清水库向城市供水工程前期工作，切实提高供水、供热、供气能力。推进新兴区垃圾处理场、三区垃圾中转站等设施建设，抓好茄子河区、种畜场排水系统和城区部分管线维修改造工程，加强大气治理，搞好七勃路两侧矸石山培土造绿。

　　强力推进节能减排工作。积极开展低碳经济试点，认真落实节能减排目标责任制，大力推进重点节能减排工程建设。实施污染物排放总量控制，严格执行环境影响评价和“三同时”制度，坚决控制产能过剩行业新上项目。继续强化高耗能行业和重点耗能企业节能降耗监测监控，严厉查处偷排和超标排放行为，确保完成“十一五”节能减排约束性指标。

　　继续加大城乡环境整治。以创建“三优”文明城市为目标，以体制机制创新为保证，以精细化、数字化管理为手段，以集中整治“农村五乱”、“城市十乱”为重点，全面改善城乡环境，努力做到城市绿化、亮化、美化，农村村容整洁。启动实施桃山水库饮用水源治理工程。

　　（五）以改革开放为根本，激发经济社会发展活力。坚持改革开放不动摇，进一步解放思想，发扬敢为人先的精神，通过深化改革破解发展难题，在扩大开放中赢得发展机遇。

　　扎实推进重点领域改革。完成市、县（区）政府机构改革，坚持精简、统一、效能的原则，科学制定“定职责、定机构、定编制”方案，进一步理顺部门关系，强化部门责任，转变政府职能。稳步推进事业单位分类改革，提高事业资源的利用效率和公益服务质量。积极推进医改试点市和公立医院改革试点，加快实现基本公共卫生服务均等化。深化农村综合改革，推行“三资”委托代理服务制。加快集体林权制度改革。积极推进财税和价格改革，深化部门预算改革。完善政银企对接机制，实现金融机构与企业互利共赢。积极支持企业上市、发行债券。

　　努力提高对外开放水平。认真落实省政府关于对俄经贸合作适应性战略调整的总体部署，加快出口基地建设，优化出口结构，培育出口品牌。发挥我市至俄罗斯国际客货运输线路的作用，进一步壮大经贸主体，扶持一批企业做大做强。加快实施“走出去”战略，支持有条件的企业到境外投资办厂，扩大境外资源合作开发、工程承包、劳务合作和农业开发。大力引进关键设备和先进技术、产业资本。

　　大力发展非公经济。认真落实国家和省关于促进非公经济和中小企业发展的政策措施，进一步解放思想，改善发展环境，加大扶持力度，推动非公经济和中小企业快速发展。积极鼓励全民创业，降低准入门槛，加强创业培训和辅导。大力实施中小企业成长工程，发挥中小企业发展专项资金引导作用和创业孵化器作用，鼓励中小企业围绕主导产业和骨干企业协作配套。坚持外商内商一视同仁，外资内资一样优惠，全力支持本地企业和民间资本投资兴业。

　　全力抓好招商引资。创新招商引资工作机制和方式方法，领导招商、专业招商、企业招商相结合。突出招商重点，围绕主导产业、替代产业、新兴产业和骨干企业招商。瞄准重点区域、重点企业、重点项目定向招商。积极承接发达地区产业转移，加强与中省直大企业和知名企业合作。注重招商引资的质量和效果。研发和储备一批市场前景好、经济效益高的大项目，提高对外招商的吸引力和成功率。积极参加各类展会招商，办好上海世博会七台河活动周。强化招商引资目标考核，严格兑现奖惩。加强对上联系，及时准确掌握国家和省投资重点，积极做好对上争取。

　　大力改善经济发展环境。严格兑现各种优惠政策，规范行政审批，清理收费项目，减少办事环节，提高办事效率。严格问责制度，打击破坏企业生产经营、阻碍项目推进的各种违法行为，克服部门主义、本位主义。加强诚信建设。

　　（六）以保障改善民生为出发点和落脚点，促进社会事业全面发展。坚持以人为本，保障和改善民生为先，切实把科学发展的成效体现在提高人民群众生活水平和质量上。今年要做好以下六项民生工程。

　　继续扩大就业和再就业。实施更加积极的就业政策，千方百计拓宽就业渠道。鼓励高校毕业生面向基层、中小企业就业和自主创业，支持退役士兵自谋职业。加强农民工职业技能培训，引导就地就近就业和返乡创业。进一步开辟公益就业岗位，帮助城镇就业困难人员和零就业家庭劳动力就业。城镇登记失业率控制在4.3%以内。

　　进一步完善社会保障体系。继续扩大基本养老、医疗、失业、工伤、生育等社会保险覆盖面，适时提高城乡低保标准，提高企业退休人员基本养老金和部分优抚对象待遇水平。适时提高城乡居民医保报销标准。力争城镇居民医保参保率达到90%，新农合参合率达到98%。

　　坚持优先发展教育事业。继续加大教育投入，改善办学条件，启动中小学校舍安全工程建设，加快农村寄宿制学校建设。推进优质教育资源共享，促进城乡、区域教育均衡发展。解决好困难家庭子女上学问题。巩固提高义务教育，加快发展高中阶段教育，大力发展职业教育，全面加强特殊教育和学前教育。加强师德师风建设，提高教育教学质量。规划建设七台河技师学院。

　　完善城乡公共卫生和计划生育服务体系。提高医疗卫生服务水平，完善公共卫生医疗服务体系，市区社区卫生服务机构全部实现标准化。落实医药卫生体制改革实施方案，引导优质卫生资源向基层转移，启动农村卫生社区化管理，实现县和乡镇医院、村标准化卫生所全覆盖。广泛宣传普及健康知识。大力发展中医药事业。做好各类传染病疫情测报防控工作。加强人口和计划生育工作。保护妇女和未成年人权益。支持残疾人事业加快发展。继续加强老龄工作。

　　 大力发展文化体育事业。积极发展公益性文化事业，加快完善公共文化服务体系，丰富群众文化生活。做好文化中心规划设计等前期工作。推进文化惠民工程。推广普及数字化电视传输。加快文化产业发展。加强公共体育设施建设，完成体育会展中心建设。推动群众性体育活动和竞技体育全面发展。承办好大型体育赛事。力争在第十二届全省运动会上取得好成绩。完善科技企业三级孵化器。搞好人才培养、引进和使用。争创国家“双拥”模范城。

　　加快保障性住房建设。加大保障性住房建设投入，完成惠民二期等3个棚改小区、47.9万平方米住宅楼建设，新开工建设3个棚改小区、53万平方米，完成实物配租廉租房200套，新增货币补贴家庭1000户。改造农村泥草房4820户、特困户危房400户，市区泥草房全部完成改造。

　　（七）以强化安全生产为着力点，切实维护社会和谐稳定。牢固树立安全发展理念，正确处理经济发展与安全生产的关系。

　　全力抓好安全生产。强化企业安全生产主体责任和政府安全监管责任。以煤矿安全生产为重点，进一步落实安全生产责任制，强力推进小煤矿关闭和瓦斯治理。深入开展道路交通、消防森防、建筑施工、特种设备、易燃易爆和危险化学品、非煤矿山以及食品药品生产流通等重点领域专项整治，开展经常性的隐患排查治理，严格现场管理和日常安全监管，切实加强班组建设，坚决遏制重特大安全事故发生。

　　强化社会治安综合治理。深入推进平安七台河建设，严密防范和依法打击各类违法犯罪活动，增强人民群众安全感。加强国防动员和后备力量建设，巩固军政军民关系。

　　进一步加强信访工作。重视维护群众合法权益，及时合理解决群众反映的问题，坚决纠正各种损害群众利益的行为。完善矛盾纠纷调处化解机制，引导群众以理性合法方式表达利益诉求。坚持领导干部接访制度。严格落实信访工作责任制。健全社会稳定预警机制和突发事件应急管理体制，有效预防、妥善处理各类突发事件和群体性事件。

　　（八）以提高行政效能为切入点，加快建设服务型政府。切实加强政府自身建设，努力建设人民满意的政府。

　　提高素质能力。坚持发展第一要务，以人为本、执政为民，以建设学习型政府为载体，认真学习实践科学发展观，不断充实现代经济、金融、科技、法律和社会管理知识，努力形成适应形势任务发展变化的知识结构，解放思想，学用结合，不断提高履职尽责、服务群众的本领。

　　加快转变职能。认真组织实施政府机构改革，健全政府职责体系，全面正确履行政府职能。畅通政府与群众双向沟通渠道，努力建设服务型政府。切实提高行政效率，推进政务公开。

　　坚持依法行政。严格依照法定权限和程序行使权力、履行职责、接受监督。自觉接受市人大及其常委会的依法监督，主动接受市政协的民主监督，加强舆论监督和群众监督。认真办理人大代表的议案、建议、批评、意见和政协委员的提案。全面推行行政执法责任制和过错追究制。坚持依法科学民主决策，健全民主制度，丰富民主形式，严格议事规则和程序。

　　转变工作作风。坚持问政于民、问需于民、问计于民，深入实际、深入基层、深入群众，真诚为群众排忧解难。大力弘扬求真务实精神，坚决反对形式主义、官僚主义，反对弄虚作假、形象工程。大力精简会议、文件，切实改进会风、文风。强化行政问责，狠抓工作落实，提高工作效率。

　　加强廉政建设。以完善惩防腐败体系为重点，严格落实廉政建设责任制，坚持用制度管权、管事、管人，确保权力规范运行。严格执行领导干部廉洁自律规定。坚决纠正损害人民群众利益的不正之风，严厉查处各种腐败行为。坚持勤俭节约，反对铺张浪费。

　　各位代表，历史是人民创造的。任何挑战都改变不了煤城儿女创造美好生活的坚定信念，任何困难都阻挡不住七台河人民加快发展的坚实步伐！光荣的使命和人民的期待给了我们搏击风浪、负重前行的强大动力。让我们更加紧密地团结在以胡锦涛同志为总书记的党中央周围，在市委的坚强领导下，深入贯彻落实科学发展观，扎实工作，锐意进取，为确保实现三年翻番的跳跃式发展目标而努力奋斗!
